# Deriving nutrient criteria to support ʽgoodʼ ecological status in European lakes: An empirically based approach to linking ecology and management

**DOI:** 10.1016/j.scitotenv.2018.09.350

**Published:** 2019-02-10

**Authors:** Sandra Poikane, Geoff Phillips, Sebastian Birk, Gary Free, Martyn G. Kelly, Nigel J. Willby

**Affiliations:** a European Commission Joint Research Centre, Directorate Sustainable Resources, Water and Marine Resources Unit, I-21027 Ispra, (VA) Italy; b Biological and Environmental Sciences, University of Stirling, Stirling FK9 4LA, United Kingdom; c Department of Aquatic Ecology, Faculty of Biology, University of Duisburg-Essen, Universitätsstrasse 5, 45141 Essen, Germany; d Environmental Protection Agency, McCumiskey House, Richview, Clonskeagh Road, Dublin 14, Ireland; e Bowburn Consultancy, 11 Monteigne Drive, Bowburn, Durham DH6 5QB, United Kingdom

**Keywords:** Eutrophication, Nutrients, Phosphorus, Nitrogen, Macrophytes, Water Framework Directive

## Abstract

European water policy has identified eutrophication as a priority issue for water management. Substantial progress has been made in combating eutrophication but open issues remain, including setting reliable and meaningful nutrient criteria supporting ʽgoodʼ ecological status of the Water Framework Directive.

The paper introduces a novel methodological approach - a set of four different methods - that can be applied to different ecosystems and stressors to derive empirically-based management targets. The methods include Ranged Major Axis (RMA) regression, multivariate Ordinary Least Squares (OLS) regression, logistic regression, and minimising the mismatch of classifications. We apply these approaches to establish nutrient (nitrogen and phosphorus) criteria for the major productive shallow lake types of Europe: high alkalinity shallow (LCB1; mean depth 3–15 m) and very shallow (LCB2; mean depth < 3 m) lakes.

Univariate relationships between nutrients and macrophyte assessments explained 29–46% of the variation. Multivariate models with both total phosphorus (TP) and total nitrogen (TN) as predictors had higher R^2^ values (0.50 for LCB1 and 0.49 for LCB2) relative to the use of TN or TP singly. We estimated nutrient concentrations at the boundary where lake vegetation changes from ʽgoodʼ to ‘moderate’ ecological status. LCB1 lakes achieved ʽgoodʼ macrophyte status at concentrations below 48–53 μg/l TP and 1.1–1.2 mg/l TN, compared to LCB2 lakes below 58–78 μg/l TP and 1.0–1.4 mg/l TN. Where strong regression relationships exist, regression approaches offer a reliable basis for deriving nutrient criteria and their uncertainty, while categorical approaches offer advantages for risk assessment and communication, or where analysis is constrained by discontinuous measures of status or short stressor gradients.

We link ecological status of macrophyte communities to nutrient criteria in a user-friendly and transparent way. Such analyses underpin the practical actions and policy needed to achieve ʽgoodʼ ecological status in the lakes of Europe.

## Highlights

• We link ecological status of lake macrophyte communities to nutrient levels.
• We establish nutrient criteria for ʽgoodʼ ecological status in shallow lakes of Europe.
• Different regression and categorical methods yield similar nutrient criteria.
• Empirically derived nutrient criteria can guide lake restoration efforts.
• This methodology can be applied to other ecosystems and indicators.

## Graphical abstract

**Figure:**
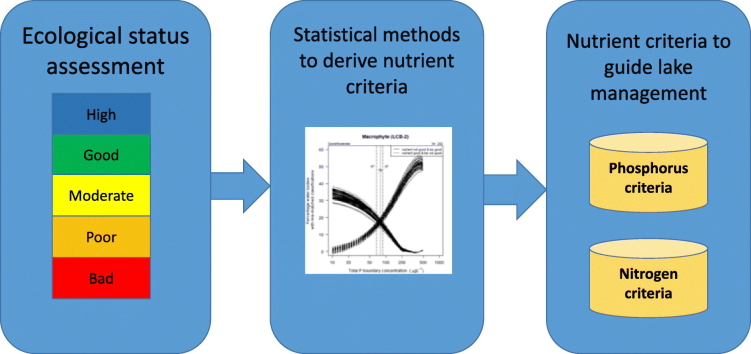

## 1 Introduction

Human activities – intensive agricultural land use, wastewater disposal and combustion of fossil fuels – have dramatically increased nutrient loading to the aquatic environment (Carpenter et al., 1998; Smith and Schindler, 2009). The rate of nitrogen input into the terrestrial nitrogen cycle has doubled since pre-industrial times (Vitousek et al., 1997), while there has been an approximately threefold increase in phosphorus inputs to the biosphere, mainly through use of fertilizers (Bennett et al., 2001). Undesirable disturbances in lakes, such as toxic cyanobacterial blooms (Carvalho et al., 2013a), loss of submerged vegetation (Sand-Jensen et al., 2000; Zhang et al., 2017), severe oxygen deficiency (Diaz and Rosenberg, 2008) and decline in sensitive fish species (Müller and Stadelmann, 2004) are commonly associated with nutrient enrichment. Therefore, eutrophication impairs ecosystem services and incurs high economic costs (Dodds et al., 2008; Le et al., 2010).

Evidence suggests that lowering anthropogenic nutrient loading to aquatic ecosystems is key to controlling eutrophication (Schindler et al., 2016; Vollenweider, 1992), but how low is ‘low’ and which nutrients to target? Nutrient management is costly and complex (Schindler, 2012) so an appropriate nutrient management strategy is critical if it is to deliver the sought-after ecological gains (Conley et al., 2009).

During the last few decades, substantial achievements in nutrient control have been made (e.g., Kronvang et al., 2005). However, improvements in the ecological status of lakes have been relatively slow, with some lakes failing to recover their original clear water state despite substantially reduced nutrient loading (Søndergaard et al., 2007). Delayed recovery has been recorded, in particular for lake macrophyte communities (Bakker et al., 2013; Eigemann et al., 2016; Jeppesen et al., 2005; Lauridsen et al., 2003). Explanations include high internal loading of phosphorus from sediments (which may last longer than 20 years; Søndergaard et al., 2003) and complex biotic interactions, especially for shallow lakes, which can switch between alternative stable states (Hilt et al., 2018; Scheffer and van Nes, 2007). As nutrient concentrations increase such lakes are more prone to switch from a vegetated to turbid state (Phillips et al., 2016), but to restore the desired vegetated clear water state, nutrient levels may need reducing to well below those at which vegetation collapsed (Ibelings et al., 2007). Setting appropriate nutrient criteria is therefore key to effective lake management.

A wide variety of approaches have been used to derive nutrient criteria (Charles et al., 2019; Dodds and Welch, 2000; Huo et al., 2017). The stressor-response approach involves modelling statistical relationships between nutrient concentrations and biological metrics (Dolman et al., 2016; US EPA, 2010). This method has the advantage of linking nutrient criteria directly to predefined ecological outcomes. For instance, in rivers, nutrient criteria are set to prevent benthic chlorophyll exceeding specific levels (Dodds and Welch, 2000), whilst for lakes, critical thresholds for cyanobacterial blooms have been used to define nutrient criteria (Carvalho et al., 2013a; Downing et al., 2001; Yuan et al., 2014; Yuan and Pollard, 2015).

However, this approach necessitates quantifying robust stressor-response relationships which in some cases has proved to be a task of daunting complexity (Borics et al., 2013; Dodds et al., 2002). Many studies have established strong empirical links between phytoplankton and nutrients (Carvalho et al., 2013b; Phillips et al., 2013), yet macrophyte-nutrient relationships are much less studied. Relationships have been established between nutrients and macrophyte metrics such as colonization depth (Søndergaard et al., 2013), total cover (Han and Cui, 2016) or trophic indices (Lyche-Solheim et al., 2013; Penning et al., 2008). However, on their own these are of little use for lake management, as different metrics can respond differently to eutrophication and re-oligotrophication processes (Pall and Moser, 2009) or responses can vary between lake types (Kolada et al., 2014). There is a need to establish stressor-response models linking nutrients and holistic assessments of macrophyte communities that integrate several measures of plant composition and abundance, and on a type-specific basis. However, the issue is complex as various lake properties, such as lake size and depth, as well as climate, will influence these criteria (Scheffer and van Nes, 2007).

In theory, waterbody-specific criteria could be developed, considering all relevant factors. However, in real-life situations, where managers must cope with restricted resources, limited data, transboundary water issues and a huge number of water bodies (Finland - 4275, Poland - 1038 and Sweden - 7232 lake water bodies; ETC/ICM, 2012) establishing broad-scale type-specific nutrient criteria is justified. These type-specific criteria also offer a high-level screening tool for prioritizing lakes ahead of more focused nutrient-management activities (Bennion et al., 2005).

The Water Framework Directive (WFD; EC, 2000) was adopted to protect and enhance Europe's water resources. It requires the ecological status of water bodies to be classified according to (1) biological elements (phytoplankton, benthic invertebrates, fish fauna, macrophytes and phytobenthos), (2) chemical and physico-chemical elements (e.g. nutrients, oxygen, transparency, salinity, temperature, and specific pollutants), and (3) hydromorphological elements (e.g. lateral connectivity). Water bodies are classified into five status categories: high (no or minor anthropogenic impact), good (slight anthropogenic impact) - which represents the required minimum goal for water management, and moderate, poor or bad. Two decades have been devoted to developing and harmonizing the biological assessment systems of EU member states (Birk et al., 2012, Birk et al., 2013; Poikane et al., 2014, Poikane et al., 2015). However, gaps remain regarding nutrient criteria, i.e. the values required to support biology of a given status. Recent analysis (Phillips and Pitt, 2016) found that the methods used to set nutrient criteria varied widely between member states, with large ranges in the nutrient values stated to support ‘good’ ecological status (GES). While variation is expected due to specific environmental conditions, large differences remain within common water body types. Moreover, the relationship between nutrients and biology that underpins these criteria is often unclear.

This study (1) establishes stressor-response models linking macrophyte status and nutrient concentrations; (2) estimates nutrient (total phosphorus and total nitrogen) criteria that support GES for macrophytes in the commonest lake types of lowland Europe and (3) compares these criteria and discusses their applicability.

Macrophyte status reveals the onset of undesirable ecological changes in productive shallow lakes, while empirically derived nutrient criteria guide the urgency, scale and design of remedial action, and serve as a benchmark for assessing progress.

We focus here on high alkalinity shallow lakes as these are commonly degraded by nutrient enrichment and are therefore among the most challenging to manage, while macrophytes play a pivotal role in their functioning and the restoration of macrophytes is therefore a common management priority (Coops et al., 2007; Søndergaard et al., 2007). The relative importance of nitrogen and phosphorus in driving eutrophication of such lakes also remains contested (Moss et al., 2012; Søndergaard et al., 2017) and there is thus a case to develop both N and P criteria in parallel.

## 2 Material and methods

### 2.1 Background

In this study, we use data from the Central Baltic region of Europe, which were collated for the purpose of intercalibrating biological metrics (Portielje et al., 2014). As required by the WFD, EU member states established biological assessment methods for macrophytes in lakes. Nine countries of Central Europe and the Baltic countries are included in this study: Belgium (Leyssen et al., 2005), Denmark (Søndergaard et al., 2010), Germany (Schaumburg et al., 2004), the Netherlands (Coops et al., 2007), Poland (Ciecierska and Kolada, 2014), United Kingdom (Willby et al., 2012), Estonia, Latvia, and Lithuania (Portielje et al., 2014; Poikane et al., 2015, Poikane et al., 2018). National assessments were expressed as an Ecological Quality Ratio (EQR) ranging from 1 (near-natural condition) to 0 (the worst possible ecological condition) and each national method was applied to the macrophyte survey data of all other countries.

National boundaries for high-good and good-moderate status were intercalibrated and harmonized between European member states using a common macrophyte metric to express each member state's boundaries on a common scale (Portielje et al., 2014; Poikane et al., 2015, Poikane et al., 2018). We used this common metric EQR and the intercalibrated class boundaries to establish relationships with nutrients from which nutrient criteria were determined. Nutrient concentrations used were annual mean total phosphorus (TP) and total nitrogen (TN) values for each lake (Table 1).

**Table 1:** Summary of data used showing lake types and range of nutrient values available.

Countries	Lake code	Lake type description	Number of lake-years		Range of values	
			For regressions	For categorical methods	For TP (μg/l)	For TN (mg/l)
Belgium Denmark Germany Estonia Latvia Lithuania The Netherlands Poland UK (Central and East)	LCB1	High alkalinity (>1.0 meq/l) shallow (mean depth 3–15 m)	87	161	8–597	0.22–6.4
	LCB2	High alkalinity (>1.0 meq/l) very shallow (mean depth < 3 m)	202	202	9–1466	0.16–11.9

### 2.2 Methods for establishing nutrient criteria

#### 2.2.1 Univariate regressions

**Fig. 1:**
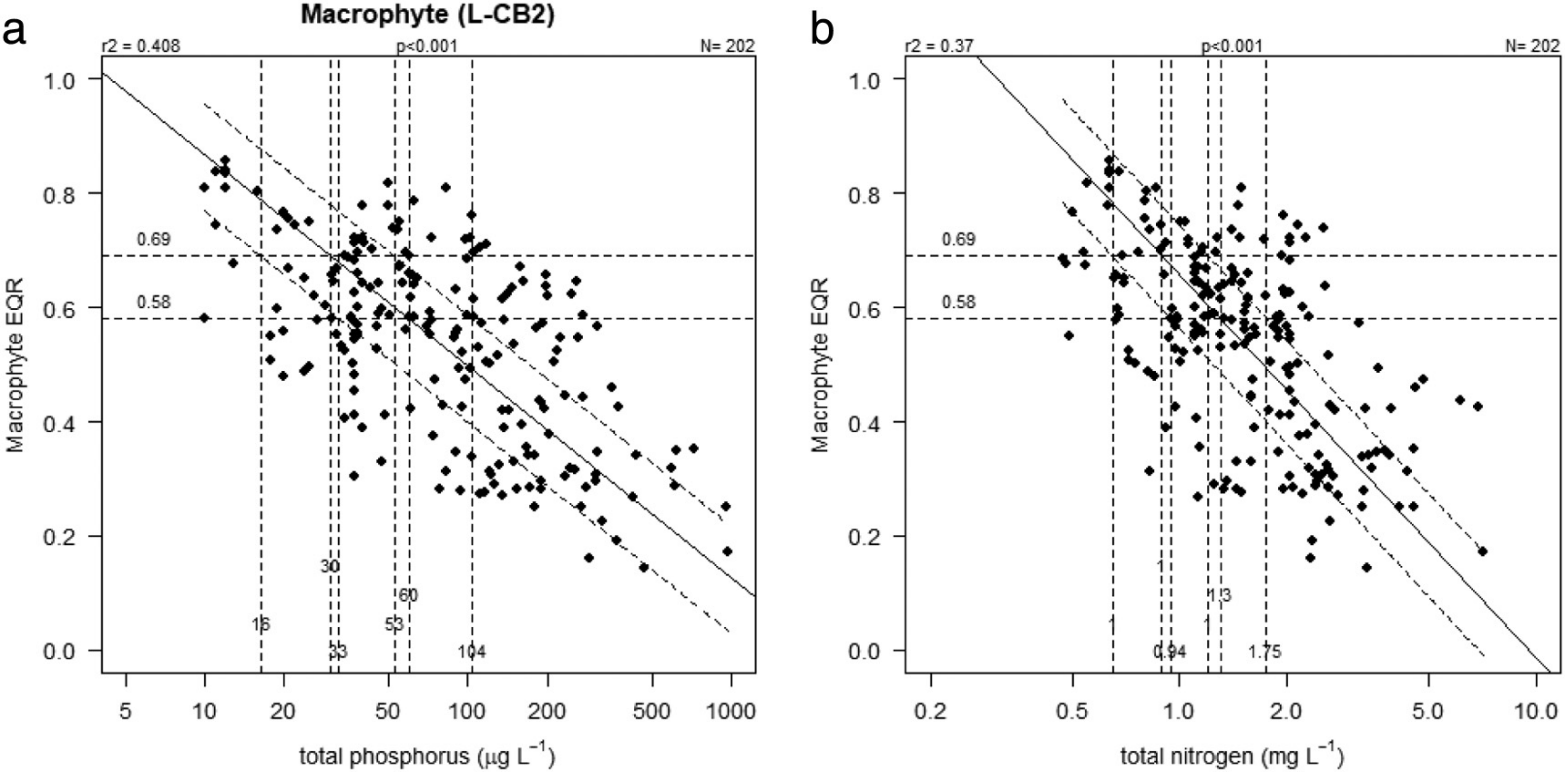
Relationship between common metric for macrophytes and a) total phosphorus and b) total nitrogen for high alkalinity very shallow (L-CB2) lakes showing high/good and good/moderate boundaries. Solid line shows type II RMA regression, dotted lines show upper and lower quartiles of residuals.

To determine nutrient values consistent with a given ecological status we compared univariate and multivariate linear regression models with a variety of categorical approaches. For the univariate regression we use Ranged Major Axis regression (RMA; Legendre and Legendre, 2012), a type II approach. Our choice of this approach reflects the significant uncertainty in estimates of both the biological EQR and mean nutrient concentrations, either of which could be treated as the independent predictor variable. We determined the linear region of the relationship by fitting a generalized additive model to visualise the relationship, and confirmed the linear region using segmented regression. After fitting univariate relationships (Fig. 1), the nutrient concentration (TN or TP) corresponding to the biological status boundary (i.e. good-moderate or high-good) was determined from the regression equation using the intercalibrated common metric criteria values.

#### 2.2.2 Multivariate regressions

**Fig. 2:**
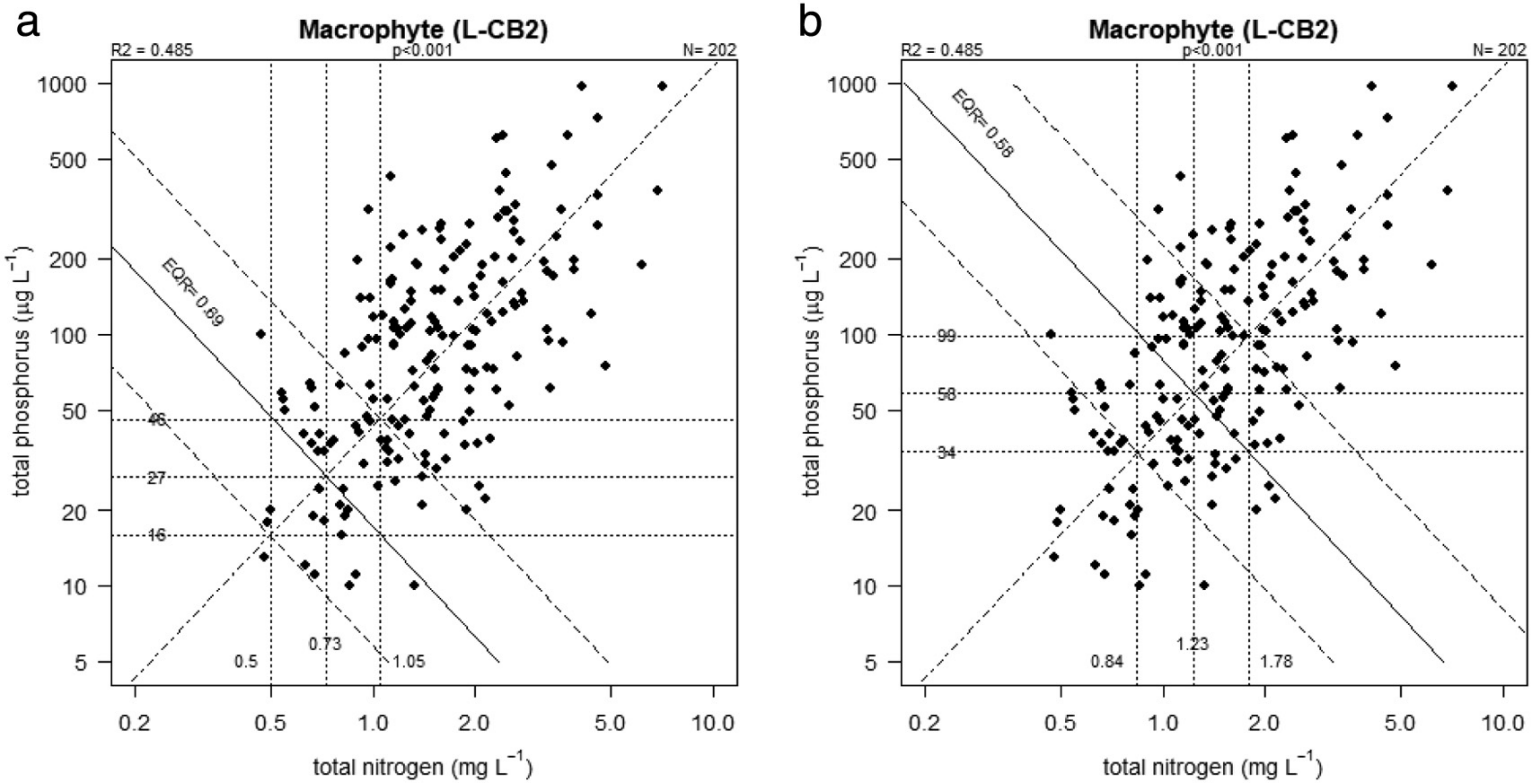
Relationship between mean TP and TN in high alkalinity very shallow lakes (L-CB2). Dotted lines show contours of predicted TN and TP concentration when macrophyte EQR is at a) high/good and b) good/moderate boundary (±25th & 75th residuals of prediction). Horizontal and vertical lines show intersection with RMA regression of observed TP and TN showing good moderate boundary concentrations.

For multivariate regression an unlimited range of potential pairs of TN and TP concentrations occur at the specified boundary EQR values. On a bivariate plot these can be expressed as contours and the values we report were those where the contour line intersected with an RMA regression fitted to the relationship between TN and TP (Fig. 2). For both univariate and multivariate approaches, we used the upper and lower quartiles of the regression residuals to determine the potential range of criteria values. We estimated uncertainty in the predicted nutrient criteria values from these quartiles, which will contain 50% of the observed data, and the most likely value associated with a particular ecological status from the fitted line.

#### 2.2.3 Categorical methods

Additionally, we used two categorical methods – logistic regression and minimising mis-match of classifications - for setting nutrient criteria. Categorical methods are less constrained by the requirements of linear regression models, they are intuitively simple to understand and may offer the best approach where relationships are weak or the stressor gradient is short.

**Fig. 3:**
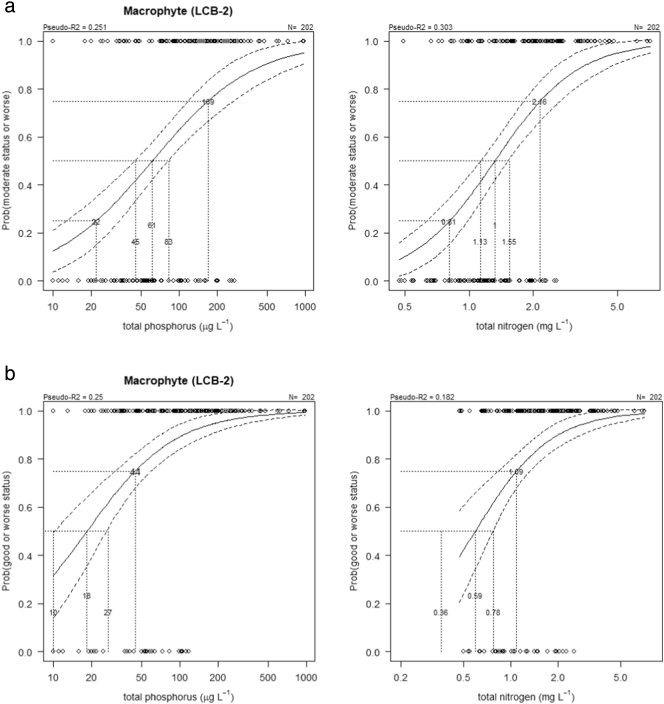
Binary logistic regression (± 95% confidence limits) between total phosphorus/nitrogen and the probability of macrophytes from high alkalinity very shallow (L-CB2) lakes being classified as a) moderate or worse, b) good or worse. Lines show potential good/moderate and high/good boundary values at p = 0.5 and intersections with fit ±95% confidence limits, and alternative values at p = 0.75 and p = 0.25 (good/moderate only) reflecting differing levels of precaution.

We fit a binomial logistic regression model to data that were classified into two groups (ʽgood or betterʼ and ʽmoderate or worseʼ - in the case of the good-moderate criteria; ʽhighʼ and ʽgood or worseʼ - in the case of high-good criteria). Nutrient boundary estimates are presented for a 50% probability of being in ʽmoderate or worseʼ status for the good-moderate criteria, or in ʽgood or worseʼ for the high-good criteria (Fig. 3). Alternative probabilities of 25% and 75% were also assessed to reflect different risks of failing to meet the desired standard.

**Fig. 4:**
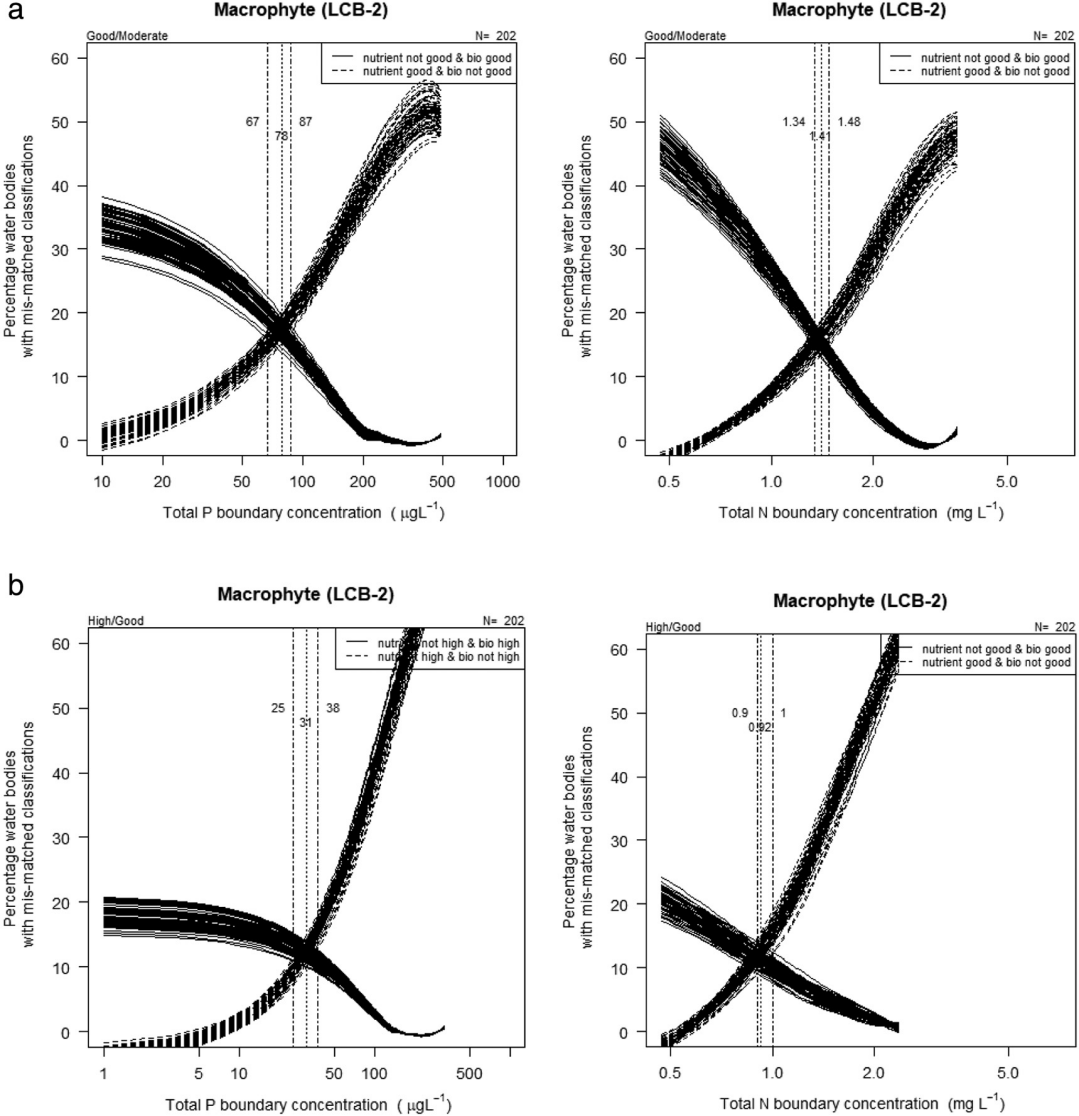
Percentage of water bodies where macrophyte or nutrient classifications for ecological status differ in comparison to the level used to set the boundary values for good/moderate or worse (top row) and high/good or worse (bottom row) for a) total phosphorus and b) total nitrogen in high alkalinity very shallow (L-CB2) lakes. Lines are loess smooths, vertical lines mark mean and range of intersections which identify the good/moderate boundary.

We estimated the nutrient concentration that gives the lowest mis-match between classifications based on biology and on nutrient concentration. Criteria values were obtained by (i) plotting the percentage of water bodies that would be at ʽgood or betterʼ status for biology but ʽmoderate or worseʼ for nutrients for different potential nutrient criteria values; (ii) overlying an inverse plot showing the percentage of water bodies where biology is moderate or worse but nutrients are ʽgood or betterʼ; (iii) estimating the point of intersection where the mismatch of classifications is minimized (Fig. 4).

**Fig. 5:**
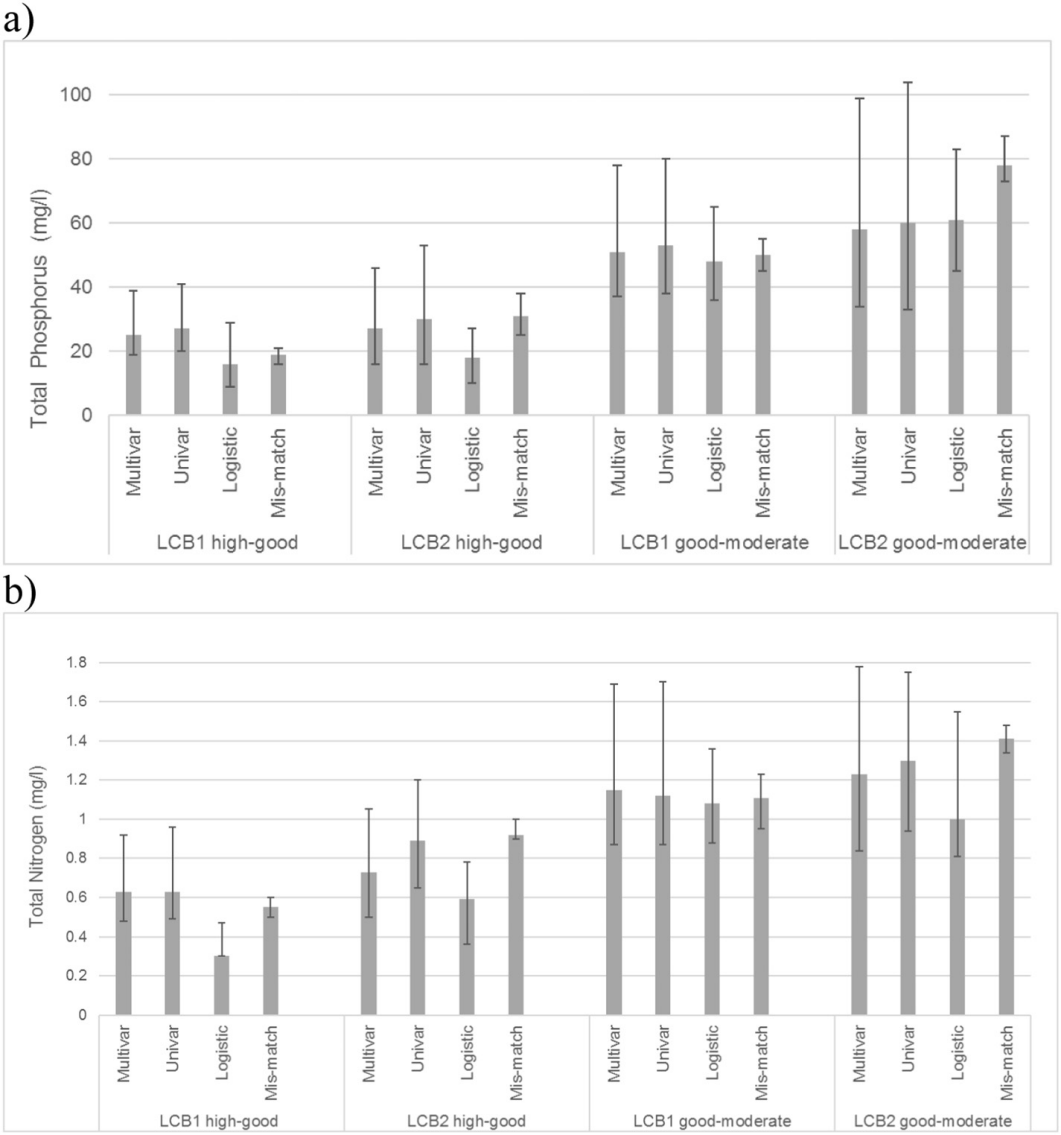
Comparison of total phosphorus (a) and total nitrogen (b) criteria for different lake types/different criteria setting methods.

We compiled the results from all of these approaches, together with their uncertainty estimates, to summarise:

• The most likely criteria, determined from the “best” regression model, where the best model was defined as the one with the highest R^2^;
• The range of potential criteria values, as defined by the upper and lower quartiles of the residuals of the best regression model;
• The range of potential criteria values derived from the upper and lower values predicted from the different regression and categorical approaches (Fig. 5).

Nutrient concentrations were log transformed prior to modelling. Statistical analyses were carried out with the R software package (R Core Team, 2016). GAM models were fitted using the mgcv package (Wood, 2010), segmented regression with segmented (Muggeo, 2009) and RMA with lmodel2 (Legendre, 2011).

## 3 Results

### 3.1 Univariate regression models

For high alkalinity very shallow lakes (LCB2), the relationship with total phosphorus predicted a concentration for the good-moderate boundary of 60 μg/l, with 50% of the data having values between 33 and 104 μg/l (Fig. 1). The univariate relationships for TN had lower r^2^ values (0.37) than those for TP (r^2^ = 0.41), predicting good-moderate criteria of 1.30 mg/l with a range 0.94–1.75 mg/l. Corresponding results for the high-good boundary were 30 (range 16–53) μg/l TP and 0.89 (range 0.65–1.20) mg/l TN.

For high alkalinity shallow lakes (LCB1) the univariate relationships for TP (r^2^ = 0.46, p < 0.001) predicted a good-moderate criteria of 53 μg/l with 50% of values ranging between 38 and 80 μg/l (Fig. S1 in the supporting information). Relationships for TN (r^2^ = 0.29, p < 0.001) produced a good-moderate criteria of 1.12 mg/l with 50% of values ranging between 0.87 and 1.70 mg/l. High-good criteria were 27 (range 20–41) μg/l for TP and 0.63 (range 0.49–0.96) mg/l for TN.

### 3.2 Bivariate regression models

For the LCB2 lakes (Fig. 2), including both TP and TN in models increased the R^2^ value significantly (R^2^ = 0.49, p < 0.001) relative to both the TP and TN univariate models (r^2^ = 0.41 TP and r^2^ = 0.37 TN). The resulting good-moderate criteria values were similar to those from the univariate models (TP 58, range 34–99 μg/l; TN 1.23, range 0.84–1.78 mg/l).

For the LCB1 lakes, including both TP and TN increased the R^2^ value (R^2^ = 0.50, p < 0.001) significantly relative to the use of TN only (r^2^ = 0.29) or TP only (r^2^ = 0.46). The bivariate regression provided good-moderate criteria values similar to those predicted by univariate regression: TP 51 range 37–78 μg/l; TN 1.15 range 0.87–1.69 mg/l (Fig. S2 in the supporting information).

### 3.3 Logistic regressions

The binary logistic regression of TP and TN on macrophyte assessments are presented in Fig. 3. Nutrient good-moderate criteria correspond to a 50% probability of being classified as ʽmoderateʼ status or worse. For LCB2 lakes (Fig. 3) these criteria are 61 μg/l TP (95% confidence limits 45–83 μg/l TP) and 1.0 mg/l TN (95% confidence limits 1.13–1.55 mg/l TN). For LCB1 lakes (S2) good-moderate criteria are 48 μg/l TP (95% confidence limits 36–65 μg/l TP) and 1.08 mg/l TN (95% confidence limits 0.88–1.36 mg/l TN) (Fig. S3 in the supporting information).

Nutrient high-good criteria correspond to a 50% probability of being classified as ʽgoodʼ status or worse: LCB1: 16 μg/l TP and 0.30 mg/l TN; LCB2: 18 μg/l TP and 0.59 mg/l TN.

### 3.4 Minimise the mismatch between biological and nutrient classification

The percent of water bodies at ʻgoodʼ or better status for biology but ʻmoderateʼ or worse for nutrients for different potential criteria values was overlain on a plot of the percentage of water bodies where biology is ʻmoderate or worseʼ but nutrients are ʻgood or betterʼ. The point of intersection of these lines reveals a concentration where the rate of mismatch of classifications is minimized. For LCB2 type the values for TP were 78 μg/l and for TN of 1.41 mg/l (Fig. 4). For good-moderate criteria in the LCB1 lake type this intersection occurred at a TP concentration of 50 μg/l and a TN concentration of 1.11 mg/l (Fig. S4 in the supporting information). This approach also demonstrates that it is possible to achieve relatively low rates of mismatch, ca 10%–20%, that may be reassuring to policy makers.

### 3.5 Comparison and summary of estimated nutrient criteria

Overall, the nutrient criteria predicted by the different methods were broadly similar within lake types and higher for the shallower lake type LCB2 (Table 2, Fig. 5).

**Table 2:** Summary of predicted total phosphorus and total nitrogen criteria values for lake types. Includes the value predicted by best model and the range defined by the 25th and 75th percentiles of the residuals of the best regression model. The range of potential criteria values derived from the different regression and categorical approaches.

Nutrient	Type	Good – moderate status criteria		High – good status criteria	
		Best model (25th and 75th percentile)	Range of criteria values	Best model (25th and 75th percentile)	Range of criteria values
Total phosphorus (μg/l)	LCB1	51 (37–78)	48–53	25 (19–39)	16–27
	LCB2	58 (34–99)	58–78	27 (16–46)	18–31
Total nitrogen (mg/l)	LCB1	1.15 (0.87–1.69)	1.08–1.15	0.63 (0.48–0.92)	0.30–0.63
	LCB2	1.23 (0.84–1.78)	1.00–1.41	0.73 (0.50–1.05)	0.59–0.92

## 4 Discussion

### 4.1 Which nutrient levels support GES for macrophytes?

Most nutrient criteria for freshwaters focus on phytoplankton, ranging from classical models linking chlorophyll-a to nutrients (Carlson, 1977; Vollenweider and Kerekes, 1980) to the more recent, addressing cyanobacteria blooms and their hazards (Carvalho et al., 2013a; Yuan et al., 2014; Yuan and Pollard, 2015). Many empirical studies describe degradation of macrophyte communities across enrichment gradients (Sand-Jensen et al., 2000) and stress the need to establish nutrient criteria to combat macrophyte decline (Scheffer and van Nes, 2007). However, few studies have suggested such criteria and then only for limited regions (Ireland; Free et al., 2016; UK; Willby et al., 2012). Without empirically-based nutrient criteria the issue of how low is ‘low’ becomes one of judgement alone.

In this study, we established stressor-response models between macrophyte assessments and nutrients and set nutrient criteria, which support GES for macrophyte communities for the major lake types of lowland Europe (Table 3). Our main findings are that:

• Significant relationships exist between macrophyte status and nutrient concentration (for TP r^2^ = 0.41–0.46, for TN r^2^ = 0.29–0.37, both p < 0.001) with multivariate models including both TP and TN having higher explained variability (R^2^ = 0.49–0.50) compared with univariate models;
• Lake depth is a key factor in determining nutrient sensitivity, with very shallow lakes (mean depth < 3 m) having greater tolerance to nutrients (Fig. 5);
• Different methods applied to the same data yielded nutrient criteria values that are broadly similar (Fig. 5) and are consistent with other studies of high alkalinity lakes (Table 3, note different lake types).

**Table 3:** Various nutrient criteria set using different approaches, including this study.

Reference	Lake type	Nutrient criteria		Approach to setting criteria
		TP (μg/l)	TN (mg/l)	
Phytoplankton				
Dolman et al., 2016	Shallow (<3 m)	41–75	0.71–1.09	Supporting GES for phytoplankton
	Polymictic (>3 m)	36–51	0.48–0.67	
	Stratified lakes of Germany	21–34	0.26–0.51	
Free et al., 2016	Irish lakes	24–31		Supporting GES for phytoplankton

Cyanobacteria				
Carvalho et al., 2013a	Medium-high alkalinity lakes of Europe	22	–	10% of lakes exceeded the WHO low risk threshold
		48	–	10% of lakes exceeded the WHO moderate risk threshold
Downing et al., 2001	Northern temperate lakes	30	–	Minimal risk of Cyanobacteria dominance
		70		40% risk of Cyanobacteria dominance
Yuan and Pollard, 2015	US lakes	25 (16–39)	0.37 (0.26–0.54)	Exceedance of WHO low risk threshold
		87 (57–130)	1.1 (0.75–1.5)	Exceedance of WHO moderate risk threshold
Yuan et al., 2014	US lakes	–	0.57–1.1	Probability of high microcystin concentrations at or below 10%
		–	0.25–0.40	Probability of high microcystin concentrations at or below 5%

Macrophytes				
Willby et al., 2012	High alkalinity UK lakes (<3 m) High alkalinity UK lakes (3–15 m)	49–66	–	Site-specific model including alkalinity and lake depth
		38–44	–	
Free et al., 2016	Irish lakes	16–19	–	Supporting GES for macrophytes
This study	LCB1 (3–15 m)	51	1.15	Predicted by best model
		37	0.87	75% of lakes reaching good status
		48–53	1.08–1.15	Range predicted by different approaches
	LCB2 (<3 m)	58	1.23	Predicted by best model
		34	0.84	75% of lakes reaching good status
		58–78	1.0–1.41	Range predicted by different approaches

### 4.2 Relationships between macrophyte communities and nutrients

While highly significant, the relationships between macrophyte status and nutrient concentration show considerable unexplained variability, reflecting the limitations of simple models to describe complex biological systems (Moss, 2008; Søndergaard et al., 2011). The explained variability (41%–46% for TP univariate models and 29–37% for TN; Fig. 1) is similar to other relationships between macrophytes and nutrients based on a variety of metrics (r^2^ = 0.34; Kolada et al., 2014; r^2^ = 0.31–0.55, Lyche-Solheim et al., 2013; r^2^ = 0.31–0.43; Penning et al., 2008; r^2^ = 0.24–0.31, Søndergaard et al., 2010; r^2^ = 0.49; Willby et al., 2012). However, the relationships are generally weaker than for phytoplankton metrics (Carvalho et al., 2013b; Dolman et al., 2016; Lyche-Solheim et al., 2013; Phillips et al., 2008, Phillips et al., 2013).

High unexplained variation in macrophyte status is not surprising as many other factors, absent from the analysis, will influence lake macrophytes. The importance of intrinsic factors, such as water body alkalinity, depth, size and colour (Søndergaard et al., 2010; van Geest et al., 2003; van Nes et al., 2002; Willby et al., 2012) is diminished by partitioning lakes into types. However, variation in these parameters within types will remain important, alongside inter-annual fluctuations in climate-related factors (Jeppesen et al., 2003). The effects of stressors other than nutrient enrichment can also add to uncertainty and influence the setting of reliable nutrient criteria, especially when interacting with nutrient stress: synergism may prompt overly protective values, while antagonism could lead to values being too relaxed (Côté et al., 2016). With only 26% of lake water bodies in Europe being affected by more than one pressure, multi-stressor effects are perhaps less relevant in lakes than rivers or transitional waters (Birk, 2019). However, hydro-morphological pressures (e.g. elevated water level fluctuations and shoreline modification) are still likely to influence macrophytes in some lakes (del Pozo et al., 2010; Mjelde et al., 2013; Radomski and Goeman, 2001). As nutrient loads reduce, macrophytes often recover slowly due to longer generation times, dispersal limitation, herbivory and high nutrient content in sediments (Bakker et al., 2013; Jeppesen et al., 2005; Lauridsen et al., 2003), and may also therefore not be in equilibrium with water column nutrient concentrations. Furthermore, nutrient thresholds in shallow lakes differ when switching to a turbid state versus returning to a clear-water one (Ibelings et al., 2007; Scheffer and van Nes, 2007). Collectively, these factors impose uncertainties which cannot be ignored when setting and using nutrient criteria.

### 4.3 N or P or both?

The nature of nutrient limitation is a perennial topic in limnology. Phosphorus is the key limiting nutrient in freshwaters, although nitrogen can also be limiting, especially in shallow lakes (Phillips et al., 2008), during summer (Dolman et al., 2016) and in highly eutrophic lakes (Søndergaard et al., 2017). Recent studies confirm that nitrogen contributes to the decline of macrophyte communities (González Sagrario et al., 2005; Moss et al., 2012; Søndergaard et al., 2017). The need to reduce only phosphorus (Schindler, 2012, Schindler et al., 2016), or both phosphorus and nitrogen (Conley et al., 2009; Dodds and Smith, 2016; Paerl et al., 2016) to mitigate eutrophication is, however, still disputed. For lakes, criteria setting has focused mainly on phosphorus (e.g., Carvalho et al., 2013a; Free et al., 2016) with very few studies addressing nitrogen (Dolman et al., 2016).

We show that multivariate models including both TP and TN have higher R^2^ values (0.49–0.50) than univariate models, thus stressing the relative importance of nitrogen and phosphorus in driving eutrophication in the shallow lakes of Europe and the advantage of considering both in parallel. Our study establishes nitrogen criteria 1.1–1.2 mg/l for LCB1 and 1.0–1.4 mg/l for LCB2 lakes (Table 3). These values exceed those set by Dolman et al. (2016) for phytoplankton but are similar to the 1.2–2.0 mg/l TN suggested by González Sagrario et al. (2005) as the critical threshold for switching from a clear to turbid state in Danish shallow lakes.

### 4.4 Why do shallower lakes have higher nutrient criteria?

The very shallow high alkalinity lakes (LCB-2) had higher TP criteria. One prominent cause of macrophyte decline in lakes is through light limitation caused by increasing phytoplankton shading, as reflected by strong relationships between macrophyte colonization depths and water transparency (Blindow, 1992). Because light declines exponentially with depth, more modest nutrient levels and algal growth can significantly alter macrophyte composition and abundance in deeper lakes. In addition, the macrophyte community in fertile shallower lakes may be resilient to expected increases in phytoplankton abundance because grazing zooplankton are buffered from fish predation by macrophyte-based refugia (Scheffer and van Nes, 2007), or because the water is shallow enough for rooted macrophytes to reach the surface. Variation in lake depths may thus be a significant contributor to the uncertainty in stressor-response relationships within lake types.

### 4.5 Which methods should be used to set criteria supporting GES?

Many approaches can be used to estimate and define nutrient criteria (Fig. 5). It is important to consider their advantages, limitations and the most appropriate way to use them because these approaches are an integral part of the ‘small print’ that accompanies any environmental criteria, nutrients or otherwise.

Large-scale stressor-response relationships provide a robust and efficient tool to estimate nutrient criteria for large groups of lakes (Phillips et al., 2008). Our study, similar to others, highlights the uncertainty in these relationships, explaining only 35–45% of the variation in the macrophyte response. While these models lack some of the precision of site-specific models calibrated for individual lakes or limited geographic areas (Carvalho et al., 2009; Willby et al., 2012) simple stressor-response models offer several advantages. Firstly, uncertainty can be explicitly quantified and incorporated into management decisions. For instance, it is possible to define a nutrient threshold at which a given percentage of lakes would achieve any level of status, measured on a continuous scale (Dolman et al., 2016; Phillips et al., 2013).

Secondly, assessment systems are often criticized for either being applicable only to limited geographical areas or not explicitly linked to stressors (Hering et al., 2010; Penning et al., 2008). This creates problems for transboundary river basin management requiring coordinated actions across states – a major issue for the EU where 60% of the territory lies in transboundary river basins (Hering et al., 2015). In the present analysis, we used data collected from lakes across Central Europe and the Baltic countries, developing models to underpin lake management within large geographical areas spanning several countries. In some cases local datasets can yield strong stressor-response relationships (Free et al., 2016) but the transferability of such relationships is unknown, while in many national datasets stressor gradients are too short or insufficient data is available to develop usable stressor-response relationships (Borics et al., 2013; Timm and Möls, 2012). We note that even in our own analyses, derivation of nutrient criteria for the high-good boundary was sometimes based on extrapolation into poorly populated regions of the fertility gradient and the confidence in such values is therefore lower, although they remain largely consistent with published studies.

Finally, water managers demand simple and transparent environmental standards (Birk et al., 2012). The stressor-response relationship provides a graphic and intuitive approach that is easily understood and applicable in data-poor conditions, provided its limitations are clearly communicated. It also serves to emphasise that criteria are not a ‘line in the sand’ but rather a zone in which the confidence of achieving a prescribed outcome (e.g. GES) varies.

However, variation in response is naturally high, reflecting the many factors that influence biology, whether stressor-related, intrinsic or stochastic (Søndergaard et al., 2010, Søndergaard et al., 2011). This may preclude the use of linear regression models to determine criteria values. Our analyses suggests that categorical methods (logistic regression or minimising of classification mismatches) will produce similar values to regression approaches when applied to the same dataset. These alternatives may be useful where uncertainty is high or dataset constraints apply, and provide an outcome that is easily understood by non-experts. Minimising classification mismatches may be advantageous where it is important politically that environmental regulation is seen to be ‘fair’ (e.g., sites are no more likely to ‘pass’ on biology but ‘fail’ on nutrients, than they are to ‘fail’ on biology but ‘pass’ on nutrients). Logistic regression on the other hand lends itself to the setting of alternative objectives (e.g. preventing deterioration, restoration of GES, protection of high status in designated sites) for which different levels of precaution are appropriate (Phillips et al., 2013; Yuan and Pollard, 2015). On the negative side, all categorical approaches are insensitive to the gradients in quality that exist within classes which may limit their application if the range of classes observed is small.

### 4.6 Recommendations for future applications

In this paper, we focus on nutrient enrichment - the most important anthropogenic pressure to lakes of Europe (ETC/IM, 2012) and aquatic vegetation. For shallow productive lakes, macrophyte communities represent an important component which have, to date, been surprisingly neglected in nutrient target setting. However, this approach can be applied also to other indicators, e.g., phytoplankton, benthic invertebrates and fish fauna which have been widely used in lake ecological assessment (Carvalho et al., 2013b; Lyche-Solheim et al., 2013; Poikane et al., 2016, Poikane et al., 2017). Our methodology is applicable to other pressures, e.g., water level fluctuations and shoreline modifications, as there is a growing (but still limited) evidence of the importance of these pressures to the lakes of Europe (Mjelde et al., 2013; Reyjol et al., 2014).

Moreover, the lessons derived from this are applicable much more widely. In this paper, we offer and evaluate a novel methodology which can be applied to other aquatic (and terrestrial) ecosystems and other indicators, whether in Europe or elsewhere.

## 5 Concluding remarks

• We present four methods to link GES to nutrient concentrations: RMA regression, multivariate OLS regression, logistic regression and minimising the mismatch of classifications.
• Where strong regression relationships are found, modelled values at class boundaries offer a reliable estimate of nutrient criteria and their statistical uncertainty. Conversely, categorical methods may be particularly useful where the level of uncertainty precludes robust stressor-response statistical models. Logistic regression is well suited to risk assessment where there is a need to assess the probability of compliance (e.g. 50% or 90% of lakes reaching ʽgoodʼ status) at different nutrient thresholds.
• Our study, in line with others, stresses the importance of controlling nitrogen besides phosphorus, in managing eutrophication of shallow lakes.
• In this study, we link ecological status of macrophyte communities to nutrient criteria in a user-friendly and transparent way. Such analyses can guide the practical actions and policy needed to support GES for macrophyte communities in the lakes of Europe.

## Appendix A Supplementary data

Supplementary figures Image 1
