# Supplementary material for: Deriving nutrient criteria to support ʽgoodʼ ecological status in European lakes: An empirically based approach to linking ecology and management
Source: Sci Total Environ. 2019 Feb 10;650:2074–84. doi: 10.1016/j.scitotenv.2018.09.350 (PMC6215087; doi:10.1016/j.scitotenv.2018.09.350)
Supplement: Supplementary file 1 — Supplementary figures [file mmc1.docx]

Supplementary Material

“Deriving nutrient criteria to support ʽgoodʼ ecological status in European lakes: An empirically based approach to linking ecology and management”


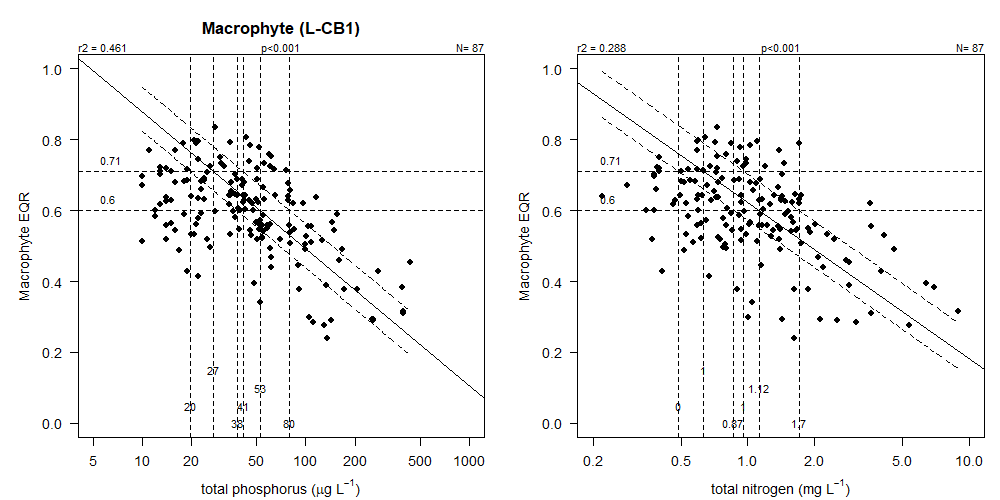


Fig S1. Relationship between common metric for macrophytes and a)total phosphorus b)total nitrogen for high alkalinity shallow (L-CB1) lakes showing high/good and good/moderate boundaries. Solid line shows type II RMA regression, dotted lines show upper and lower quartiles of residuals.


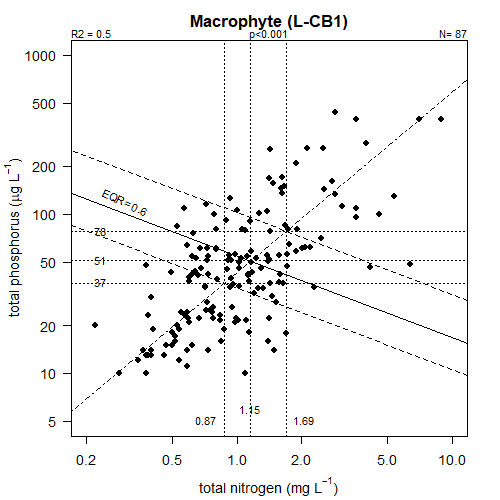

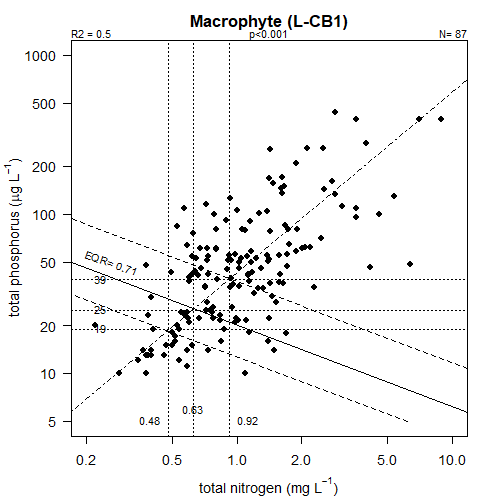


Fig S2. Relationship between mean TP and TN in a) high alkalinity shallow lakes (L-CB1). Dotted lines show contours of predicted TN and TP concentration when macrophyte EQR is at a)good/moderate and b)high/good boundary ( ± 25^th^ **&** 75^th^ residuals of prediction). Horizontal and vertical lines show intersection with RMA regression of observed TP and TN showing good moderate boundary concentrations.


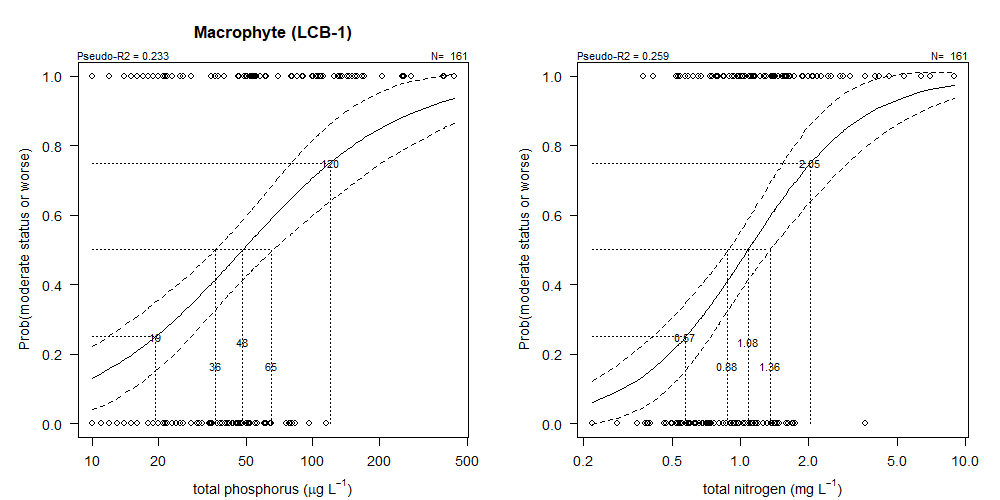

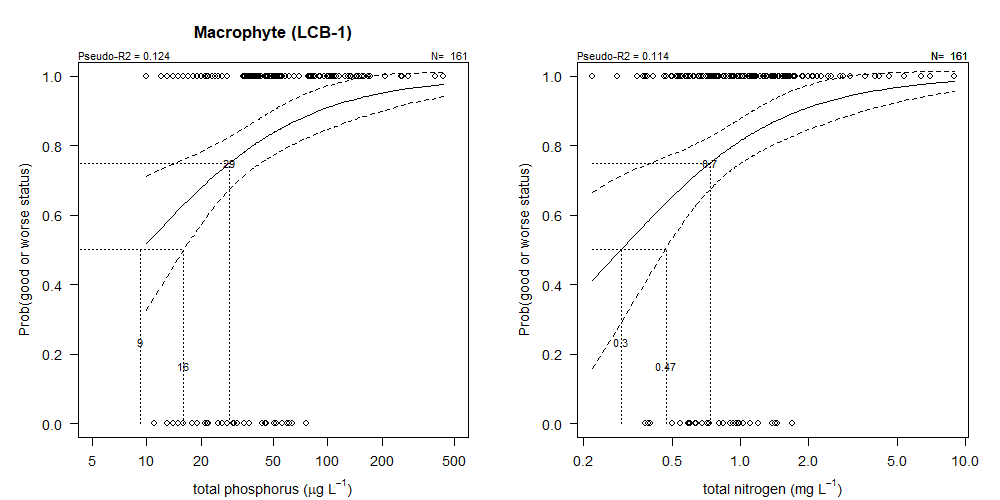


Fig S3. Binary logistic regression (± 95% confidence limits) between total phosphorus/nitrogen and the probability of macrophytes from high alkalinity shallow (LCB-1) lakes being classified as moderate or worse (top row) and good or worse (bottom row). Lines show potential good/moderate and high/good boundary values at p values of 0.5 and intersections with fit ± 95% confidence limits and where appropriate alternative values at p values 0.25, 0.75.


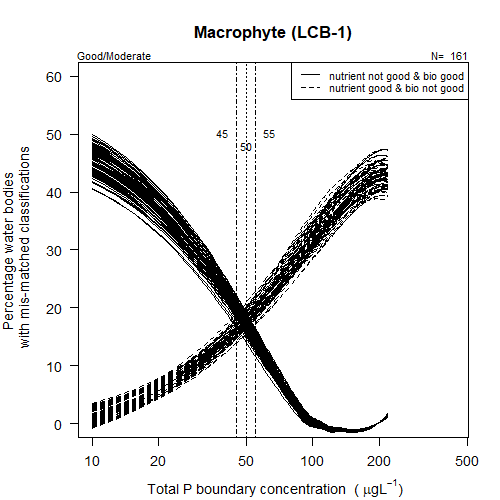

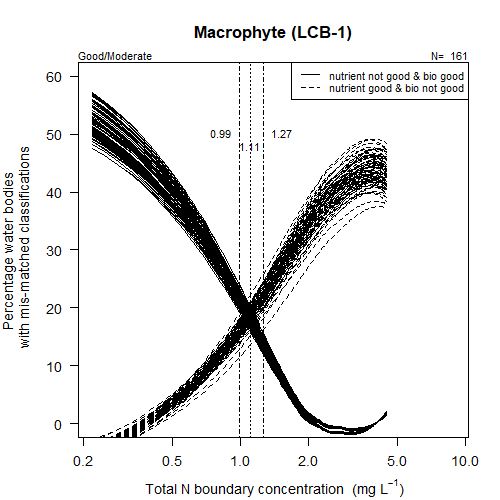

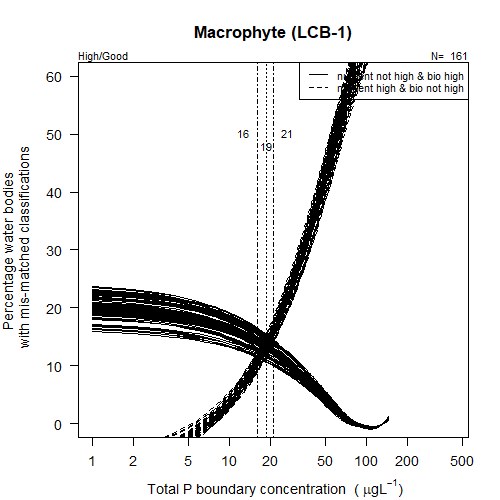

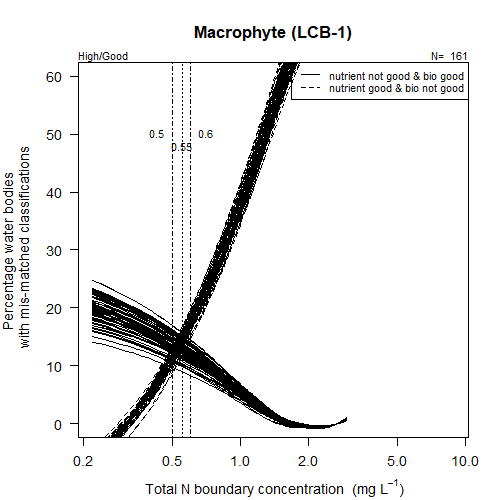


Fig S4. Percentage of water bodies where macrophyte or nutrient classifications for ecological status differ in comparison to the level used to set the boundary values for high/good or worse for total phosphorus/nitrogen in a) high alkalinity shallow (L-CB1 and b) high alkalinity very shallow (L-CB2) lakes. Lines are loess smooths, vertical lines mark mean and range of intersections which identify the good/moderate boundary.
